# Getting real in interprofessional clinical placements: patient-centeredness in student teams’ collaborative learning

**DOI:** 10.1007/s10459-022-10182-y

**Published:** 2022-11-07

**Authors:** Catrine Buck Jensen, Bente Norbye, Madeleine Abrandt Dahlgren, Anita Iversen

**Affiliations:** 1 Centre for Faculty Development, Faculty of Health Sciences, UiT The Arctic University of Norway, Tromsø, Norway; 2 Department of Health and Care Sciences, Faculty of Health Sciences, UiT The Arctic University of Norway, 9037 Tromsø, Norway; 3 Department of Health, Medicine and Caring Sciences, Linköping University, Linköping, Sweden

**Keywords:** Clinical placement, Collaboration, Interprofessional education, Patient-centered care, Student team, Thematic analysis

## Abstract

Collaboration between healthcare providers helps tackle the increasing complexity of healthcare. When learning teamwork, interprofessional students are expected to work patient-centered; recognizing the patient’s expertise and partnering with them. Research on interprofessional education (IPE) for undergraduates has illuminated learning outcomes, organization of learning activities, change in attitudes, etc. But, we know little about the interaction between patients and interprofessional student teams. This study aimed to explore how interprofessional student teams and patients interact in interprofessional clinical placements. With a focused ethnographic approach, participant observation and qualitative interviews were conducted in two contexts; a physical and an online arrangement. Central ideas in Goffman’s dramaturgy constituted a theoretical lens. A reflexive thematic analysis generated three themes: (1) Preparing safe and comfortable encounters with patients, (2) Including and excluding the patient in the encounter, and (3) Adjusting to the patient's situation. We identified students’ intentions of patient-centeredness when preparing encounters, but patients did not always feel included and listened to in encounters. After encountering patients, student teams adjusted their teamwork, by changing the team composition or the planned clinical interventions to better meet the patients’ needs. Notably, team-based patient encounters led to a different view of the patient, their health issues, and how to collaborate. Our findings can inform educators of the importance of addressing patient-centered care in interprofessional learning arrangements. Today, clinical interprofessional placements may not exploit the potential for learning about patient-centeredness. A thematization of this, e.g., in supervision in future clinical placements can ensure an enhanced focus on this in interprofessional teamwork.

## Introduction

Health professionals are expected to work interprofessionally with their peer providers as they are confronted with complex patients that require integrated, long-term care and treatment (World Health Organization (WHO), 2010, 2016). Professional health education is encouraged to train future healthcare providers on individuals’ varied healthcare needs (Frenk et al., 2010; WHO, 2010). To tackle the complex challenges that aging, chronic diseases, mental health issues, and non-communicable diseases, e.g., cancer, cardiovascular disease, and diabetes, can cause, health professionals need to be educated and prepared differently. A recent WHO-competency framework on universal health coverage accounts for competencies within six domains, including people-centeredness, decision-making, communication, collaboration, evidence-informed practice, and personal conduct (WHO, 2022). The goal is to guide the standards for education and practice to achieve a better quality of health care services, especially in primary care, where an increasing part of health care will be delivered in the future (WHO, 2022).

**Fig. 1:**
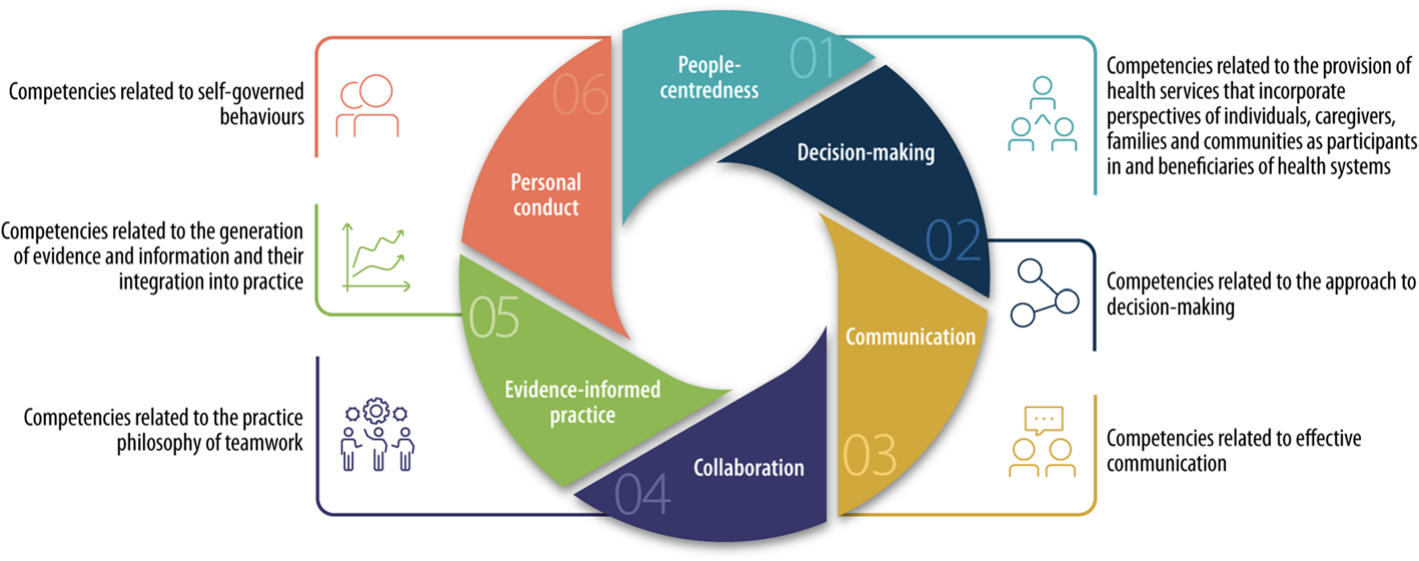
Competency domains within the Global Competency and Outcomes Framework for UHC (WHO, 2022 p. 13)

Interprofessional education (IPE) and collaborative practice are recognized as potential routes for improving the quality of healthcare service delivery (Berwick et al., 2008). IPE occurs when workers or students from two or more professions learn with, from, and about each other to improve collaboration and the quality of care and services (Centre for the Advancement of IPE, 2016). In Norway and many other European and western countries, legislation regarding healthcare services, patients’ rights to be involved in decisions concerning their care, and treatment in healthcare services have been claimed. However, this has not been dealt with thoroughly in health professional education. The 2015 Vancouver statement on “The patient´s voice in health and social care professional education” has emphasized the importance of this issue in education. The statement aims to enhance patient involvement not only in a uni-professional manner but also in interprofessional learning, as “opportunities are often missed to expand patient involvement in education beyond individual professional programs to promote team-based education and care” (Towle et al., 2016, p. 21). One priority was to “facilitate a more holistic approach to patient partnerships and teamwork” (Towle et al., 2016, p. 22). This study explores this by delving into undergraduates’ collaborative learning with patients in interprofessional clinical placements (Fig. 1).

Clinical placements are “the ideal learning environment for developing skills conducive to collaborative practice” (Hilton & Morris, 2001, p. 173). In the clinical setting, patients who have sought care may be “participating in learning by virtue of student participation in those care relationships” (Rowland et al., 2019, p. 606). Interprofessional clinical placements for undergraduates were initiated two decades ago in Linköping, Sweden (Dahlberg et al., 2020; Wilhelmsson et al., 2009). A similar organization (Oosterom et al., 2019) and numerous unique arrangements in communities and hospitals worldwide (Jensen, et al., 2022) is also found. Interprofessional clinical placements enable learning not only between interprofessional students but also between the interprofessional students and patients (Bleakley & Bligh, 2008; Rowland et al., 2019). Clinical placements allow patients in various settings with different experiences to contribute to students’ professional and interprofessional development (Rowland et al., 2019).

Thistlethwaite and Moran (2010) showed how “the patient” is central to learning outcomes for IPE. Recognizing patients’ needs, understanding the patient perspective, and including the patient as a partner within interprofessional teams were some of the themes identified (Thistlethwaite and Moran, 2010). These are all part of patient-centered care (PCC), which represents the transition from a paternalistic relationship between a doctor and a patient to an equal relationship in which the patient holds expert knowledge of their own life and health situation (Berwick, 2009). Ideally, patients will have more agency in what healthcare interventions should be implemented for their concerns. Consequently, patients and health professionals can create personal and individualized care and treatment paths (Berwick, 2009) in which the patient’s wishes are honored but not mindlessly enacted (Epstein & Street, 2011). The goal of PCC is to contribute to “a functional life for the patient” (Eklund et al., 2019 p. 8) through building emphatic and respectful relationships where the health practitioner facilitates shared decision making and a holistic focus for the individual (Eklund et al., 2019).

Studies on IPE in clinical placements where patients are included are numerous. However, the interaction between students and patients, including the patients´ role, has not been explored sufficiently (Jensen et al., 2022). Examples are studies that refer only to the patient’s diagnosis (e.g., “orthopedic patients”; Hallin & Kiessling, 2016) or in more general terms (e.g., “nursing home patients”; Baerheim & Raaheim, 2020). Some studies describe the interaction between patients and interprofessional student teams (Damsgård et al., 2018; Kent et al., 2016a, 2016b), while others have provided a more detailed description of the content in meetings between interprofessional students and service users (Ciccone et al., 2013). More extensive insight into patient and interprofessional students' interactions is however needed. An interesting aspect of said interaction is an exploration of how the placements can promote students’ interprofessional learning and collaboration, and patient-centeredness. The latter is considered a feature of all learning domains in competency frameworks on IPE (Interprofessional Education Collaborative Expert Panel (IPEC), 2016).

This study aimed to explore interprofessional student teams and patients’ interactions in clinical learning arrangements.

## Theoretical framework

The study's theoretical framework draws on concepts from Erving Goffman´s dramaturgical analysis (Goffman, 1990). Goffman’s work emphasizes micro-social interaction, that is, how individuals interact with each other and construct meaning in everyday life. An analytical focus on micro-social interaction allows a new gaze on what goes on in interprofessional clinical placement between students and patients in different contexts.

Goffman argues that human interaction in day-to-day life is controlled and staged, so we always strive to make the best possible impression on others. He puts this in parallel with actors on a stage and claims that humans continuously use impression management to be perceived the way they want (Goffman, 1990). As humans, we enter various *roles* depending on the situation in which we find ourselves. A role is a pattern of behavior related to a person's social status in a situation. When interacting, there is a shared reality between the actors; for example, in a classroom, some perform in student roles, and some serve in the teacher role. If roles are switched, the interaction would probably be disturbed, and new ways of interacting would be formed (Goffman, 1990).

Activities that individuals participate in during a limited period before a particular set of observers are considered a *performance* (Goffman, 1990)*.* According to Goffman (1990), performances are controlled and staged to manage the impressions that the performer wants the audience (one or several persons) to perceive. This type of performance happens in what he calls *frontstage*. In our case, this would correspond to the phase where students deliver interprofessional collaboration with the patient. At the frontstage, an individual will present himself following the expectations of the situation and try to live up to their role.

*Backstage*, the performer retreats from the audience and public gaze and can lower their shoulders and not be on display (Goffman, 1990). Moreover, backstage would, in our case, correspond to moments where students meet and reflect, formal or informal, either before a frontstage performance or after. Backstage performers may address each other in a different and more casual language or behavior than frontstage performers. Backstage is often where the audience is not permitted (Goffman, 1990).

In Goffman’s theory, the individual is the starting point, but he also shows how individuals are related to each other in a performance. Through the term *team,* he refers to “any set of individuals who cooperate in staging a single routine” (Goffman, 1990, p. 6). Members of the team are in a critical relationship consisting of two components: reciprocal dependency and reciprocal familiarity: First, each member must rely on their teammates and trust that they will behave to achieve the team’s best performance. Second, team members need to develop familiarity with each other, which includes letting the team performance take precedence over the individual frontstage performance. The italicized terms above will further inform our analysis.

## Methodology

The study is designed as a qualitative collective case study; it includes multiple cases and focuses both within each case and across cases (Kekeya, 2021 p. 35). The common methodology of the case studies is a focused ethnographic approach inspired by Andreassen et.al. (2020) and Higginbottom et.al. (2013). This approach is well-suited for research on health professional education, and a focus on particular issues in learning arrangements can be expedient (Andreassen et al., 2020). Beyond this, focused ethnography is pragmatic, as topics are often pre-selected, and data generation is conducted within a given timeframe or event (Higginbottom et al., 2013). The decisions to focus on students´ interaction with patients in interprofessional learning arrangements were decided before the empirical studies.

### Study contexts

The study contexts comprise two different arrangements for students' interprofessional learning. Common for the contexts is interprofessional undergraduate students encounters with patients in clinical settings.

The first learning arrangement is physical, situated at a community health center, where students do their clinical placement. The second learning arrangement is students’ online encounters with patients in different clinical settings, such as a hospital ward and an assisted-living facility (see Table 1). The online arrangement was initially physical but was digitalized due to the Covid-19-pandemic; however, after Covid restrictions ceased, the arrangement is offered either physically or digitally, by the student's choice.

**Table 1:** Overview of the multiple sources in our data material

	Length	Health care context	Sample	Method for generating data
Physical arrangements	28 h	Community Health center	Students; Patients	Participant observation
	1 h 10 min	Community Health center	Students	Focus group interview
	25 min	Community Health center	Patient 1	Individual interview
	14 h	Community Health center	Students; Patients	Participant observation
	1 h 20 min	Community Health center	Students	Focus group interview
	33 min	Community Health center	Patient 2	Individual interview
Online arrangements	20 min	Assisted-living facility	Patient 3	Participant observation
	25 min	Assisted-living facility	Patient 3	Individual interview
	1 h 15 min	Hospital ward	Students, Patient 4	Participant observation
	34 min	Hospital ward	Patient 4	Individual interview
	1 h 8 min	Hospital ward	Students	Focus group interview

In the community health center, patients were admitted from a regional hospital or their homes, either with a plan to return home or await long-term care, e.g., in a nursing home. Interprofessional students participated in learning arrangements for two to four days in their final year. Multiple student teams consisted of 5–6 students; in the first observation period, nursing students in the teams shifted after two days. Nevertheless, each team oversaw 2–3 elderly patients with complex and chronic health issues. Student teams were encouraged to collaborate interprofessionally by providing daily care for patients, conducting holistic health assessments, and different kinds of consultations. The teams worked concurrently and had their workspace for preparations and debriefings. Students were expected to write a collective interprofessional journal summary, including their observations and suggestions for further care. Interprofessional supervisors were present in many teams´ preparations and post-encounter meetings. Uniprofessional supervision was provided if needed.

In the online encounter, the interprofessional student teams consisted of 4–6 students. Each team met one patient in a different clinical setting. The learning arrangement was estimated to last approximately eight hours. One team met a patient living in an assisted-living facility, and the other met a patient admitted to a local hospital due to an infection. Health professionals on site preselected patients. As a starting point, students were instructed to conduct an interprofessional screening with the standardized question, “What matters to you?” (Barry & Edgman-Levitan, 2012). Subsequently, they co-wrote an interprofessional care plan to be assessed by a lecturer with a pass/fail grading. The team also had to include an evaluation of the team’s work process. Arrangements in this context were intended to be carried out entirely digitally; however, in one of the two observed cases, the clinical coordinator invited a couple of students to be physically present at the hospital while the rest of the team was present online. Supervision was not provided.

In both contexts, the teams independently planned and structured meeting(s) with the patients and did not follow any procedure to organize the encounter.

### Participants and recruitment

Participants were recruited by purposeful sampling (Patton, 2002) either from being a student in an interprofessional team (*n* = 37) or a patient (*n* = 5) interacting with interprofessional student teams. A breakdown of the different students´ professions is shown in Table 2. Coordinators of the learning arrangement recruited students based on who was attending the clinical placement at the specific period of observation. Health personnel employed at the healthcare facility recruited patients based on their suitability for the student teams to learn from (e.g., health complexity, issues for all professions to grasp) and their ability to understand participation in the research.

**Table 2:** Breakdown of student teams´ professional composition

	Physical arrangements					Online arrangements	
	Team 1	Team 2	Team 1	Team 2	Team 3	Team 1*	Team 2
Nursing	*n* = 3	*n* = 2	*n* = 3	*n* = 3	*n* = 2		*n* = 1
Medicine	*n* = 1	*n* = 1	*n* = 1	*n* = 1	*n* = 1		–
Physiotherapy	*n* = 1	*n* = 1	–	–	*n* = 1		*n* = 1
Occupational therapy	–	–	–	*n* = 1	–		*n* = 1
Pharmacy	*n* = 1	*n* = 1	*n* = 2	*n* = 1	*n* = 1		–
Social work	–	–	–	–	–		*n* = 1
Biomedical lab.sci	–	–	–	–	–		*n* = 1

The student team that met Patient 3 (see Table 1) declined the invitation to participate in a focus group interview because of exams. Consent to use field notes from the observation of the team was not obtained; thus, the observational data were excluded. Nevertheless, Patient 3 participated in a telephone interview and expressed his views on the encounter.

In the community health center, a purposive sample of students representing a diversity of professional programs across the teams was interviewed. In the online encounter context, the whole team was interviewed except for two students who could not attend.

### Empirical studies

Data was generated through participant observation and interviews with students and patients (See Table 1). The first author (CBJ; Ph.D. student, RN) conducted observations and interviews in both contexts.

In the community health center, team meetings, encounters with patients, and interprofessional supervision sessions were observed. When appropriate, CBJ asked students to elaborate on their actions to understand the different situations better.

In the online encounter, CBJ joined the students on Zoom and observed the interprofessional student teams´ encounters with patients and their subsequent team meetings. CBJ had her web camera turned on but did not ask elaborate questions during the online sessions. Here the interviews were used to gain a better understanding.

Semi-structured qualitative interviews were conducted after participant observations. Focus groups with students were conducted physically and through Microsoft Teams (See Table 3). Patients participated in individual interviews, either physically or via telephone. Interview guides were developed to indicate the themes of interest. The interaction between interprofessional students and patients constituted the starting point of the interviews.

**Table 3:** Breakdown of health professional students within the different focus groups

Physical arrangements	2 medical, 2 nursing, 1 pharmacy, 1 physiotherapy
	1 medical, 2 pharmacy, 3 nursing
Online arrangement	1 occupational therapy, 1 social work, 1 nursing

### Data analysis

As our study included diverse datasets from different contexts, we found that reflexive thematic analysis (TA) allowed for a flexible cross-case approach that made it possible to identify themes and patterns across the datasets.

Comprehensive field notes and interview transcripts were imported into the qualitative data analysis software NVivo (QSR International, 1999).

A six-phase TA process (Braun & Clarke, 2020, 2022) commenced about six months after finalizing the data generation. TA was conducted with an inductive approach guided by Braun & Clarke´s (2020) understanding of this as identifying meaning “grounded in the data, rather than pure induction” (p. 331). During analysis, we iterated between the different phases and between the field notes, interview data, and theory.

Jot notes from fieldwork were rewritten into comprehensive field notes (Emerson et al., 2011) and interviews were transcribed verbatim. As part of the familiarization process, the first phase in TA, CBJ immersed herself in interviews and field notes within each case. A data analysis workshop with an extended international research group was also conducted to kick-start the analysis process with different perspectives.

The data were subsequently coded inductively and semantically with participant-driven codes to capture the participants´ explicit meaning (Braun & Clarke, 2019, 2022). Initially, field notes, transcripts from focus groups, and individual interviews across cases were grouped and coded. Candidate themes were developed, reviewed, and refined through a creative process in the research team. Patterns related to happenings in the student teams and happenings in the patient encounters were identified. At this point in the analysis, it became apparent that the patterns we found could be understood considering Goffman’s dramaturgy. We used Goffman to understand how and when patient focus occurred and how this influenced the team members´ interaction with each other and the patient. Further analyses generated three overarching themes: (1) preparing safe and comfortable encounters with patients, (2) including and excluding the patient in the encounter, and (3) adjusting to the patient’s situation (see Table 4). Excerpts from field notes and interviews are highlighted in the findings by citation marks or with a block quote.

**Table 4:** Examples of data extract, data source, codes, subthemes, and overarching themes

Data extract (data source)	Codes	Subthemes	Overarching theme
*“It was reassuring that the nursing students were familiar with the facility and electronic patient record system, and also knew the patients a bit” (Focus group)*	Nursing students’ familiarity was reassuring; Spent time exploring the patient’s health issues	Student preparations and planning	Preparing safe and comfortable encounters with patients
*Members from team 1 are standing a bit spread in the corridor after their preparation-meeting. The medical student signals that they are going to see one of their patients. CBJ asks to tag along with the team. The medical student knocks the patient´s door and goes in, we can hear that she asks if it is okay for the patient that the entire team and the researcher comes to see her. The patient answers that it is ok. (Field notes)*	Relationship with the patient; Interaction between students and patient; Facilitating a comfortable encounter; Respect for the patient	Ensuring safety and comfort for the patient	Preparing safe and comfortable encounters with patients
*“I want students to be curious, that they listen. And the ones I met were inquisitive, they were very nice people” (Individual interview)*	Positive aspects of the encounter; Students listened to patient	Patients’ expectations and experience	Including and excluding the patient in the encounter
*“We did become aware of, at least that one time, that we asked many closed-ended questions to the patient and that we may have to focus a bit more on open-ended questions” (Focus group)*	Interaction between students and patient; Asking patient’s questions; Patient encounter	Questions-inclusive and exclusive	Including and excluding the patient in the encounter
*“When I participated in the morning routine, I see that it takes time, it is not given that the patient wants to get up, so there is more to the picture than just what I think about” (Focus group)*	Patient encounter, Picture of patient is different after encounter, Positive aspects of IPE	Students experience of encounters	Adjusting to the patients’ situation

### Ethics

This study was approved by the Norwegian Centre for Research Data (no. 831589). All data were collected following the Declaration of Helsinki (World Medical Association, 2020) and the Ethical Guidelines for Educational Research (British Educational Research Association, 2018). Participants provided written consent before data generation and could withdraw from the study at any time.

### Findings

In our analysis, we have focused on two actors; patients and students. We have assumed that the student teams were already established when using Goffman as our lens to understand the interactions. Consequently, the analysis did not focus on the students' interactions when establishing their teams.

We found that student teams develop a joint backstage when focusing on one or several patients. When teams encounter patients, they perform frontstage *together* with the patient. When ending the encounter, the patient, and the team withdraw to their backstage. This movement between front and backstage could either happen as a unique episode (in the digital context) or multiple times (in the physical context).

In the following section, we provide empirical examples from our analysis and show how Goffman’s dramaturgy can shed light on how interprofessional student teams and patients interact.

### Preparing safe and comfortable encounters with patients

Across contexts, students were instructed to prepare for encounters with one or several patients. When preparing, students were initially interested in each other’s professional perspectives. Still, they switched focus from themselves to the patient and their health issues, and the students different professional views were integrated into their talk about the patient.

The teams had a respectful tone when talking about the patient and upheld their professional roles, even if they, at this point, did not interact directly with the patient. Information obtained from the electronic health records (EHRs) about patients’ goals and wishes was repeatedly discussed during preparation. Despite the patient not being physically present backstage, they became present through the students’ interactions with each other and the her and the information visible on “the widescreen”. The patient played a role backstage without being aware of it.

In the community health center, students asked questions like “What is the plan now?” “Can we call this a plan?” and “Can we take a recap of what we agreed on?” in several cases. The teams agreed on what they would do, who was asking questions, what questions each professional student needed answers to, how many would see each patient, and what professions would be favorable to have in the meeting. They agreed on who would play what role in the performance that would take place frontstage with the patient. This was also reported by students in the online arrangement, talking about how they juxta positioned their questions to structure the meeting. However, the students in the online arrangement felt it challenging to prepare the encounter as they had no information about the patient besides where they were located. The nursing student even talked about how their team prepared to improvise: “We improvised a bit, as our goal was to find out what mattered to her and what was important for her, we felt that we could follow up on what she said [and make her elaborate on those things]”.

### Including and excluding the patient in the encounter

In several observed encounters in the community health center, students obtained consent and provided information about the student team before entering the patient’s room. This was reported to provide safety and ensure a comfortable encounter for the patient. Through this action, the students invited the patient to play a part in directing the encounter. It also made it possible for the patient to prepare in their backstage before the student team entered.

The patients were the center of attention in all encounters across contexts. The students often prepared and asked the patient a series of questions. Questions mainly had a medical (or bodily) focus, relating to the patient's perception of their health condition and health-related issues. Patients answered accordingly but expressed in interviews that these kinds of questions did not always invite them to tell what was important to them. Two patients reported that they felt there were many unnecessary questions and few questions regarding their background and history. Both expressed a wish to ask questions themselves but did not get an opportunity to do so. The patient who met the student team entirely digitally felt that he did not have the chance to tell the student team about his pre-function and repeated this several times throughout the interview with CBJ.

One focus group participant expressed concerns that patients felt pressured to give “the right answers” to the student's questions. She also characterized many questions as leading and not open for the patient to tell their own story or reflect. One team in the physical context asked a patient, “What matters to you?” but the medical student involved in the encounter expressed that it was difficult to grasp the answer as the patient talked about other issues. This question was also central in the assignment for encounters in the online arrangement; the perception of having the chance to speak about themselves varied between the two patients. One felt that she got a chance to tell the student team “everything”, while the other patient, as already mentioned, thought he did not have the opportunity to tell them what was important to him.

### Adjusting to the patients’ situations

After meeting the patient, the teams adjusted in several ways: They adapted to the patient's issues and the team’s professional composition when possible. Plans were changed according to the information the students obtained in the patient encounter, and the preconceived pictures that some students expressed they had of the patients were adjusted.

When returning backstage to their meeting location (either physical or online), team members shared their perceptions and observations of the patients. Each team member and the team were somewhat forced to explicate the competencies they possessed and what they lacked concerning the patients’ goals and wishes. The student team adjusted their work processes following their encounters with the patients. In the community health center, student teams on several occasions requested help from other teams with a different professional composition that could contribute to a broader understanding of the patient; for example, when a pharmacy student contributed to a comprehensive drug review for one patient, or when a physiotherapy student contributed to the physical assessment of a patient with members from the student team. In contrast to the online arrangements, the teams in the community health center encountered patients several times during their placement which made the adjustments in the team composition possible.

Patients’ health issues were still the main focus of the teams and the central point of discussion. Students thoroughly assessed patients’ situations after encounters, and some expressed that meeting patients also led to adjustments in their preconceived picture. A pharmacy student claimed,

“(…) When we sat down to read and prepare the first day and read medical records and such, then we got a picture of how the patient was, the condition … But when we visited them [the patients] then, it was like, ‘Okay, that's not true’—what we had imagined. So, the whole picture must be included for the best possible treatment.”

Students suggested different measures to improve patients’ health status, including specific examinations, health screening, or more abstract measures, such as identity preservation. Student teams in the physical context revisited the EHR to confirm patients' statements during the encounters. Students in the digital context continued their interaction online but were forced to repeat and recall what the patient had expressed in the frontstage performance without accessing the patient’s EHR.

Students seemed to become more aware of their peers’ knowledge and perspectives after meeting the patient and similarities or differences compared to their roles. Some students with limited clinical experience expressed gratitude to fellow students (on several occasions, nursing students) who helped them feel safe with the patient. Nevertheless, as uncomfortable as it may have been, the frontstage performance was expressed as necessary to gain insight into the different patients’ spheres and learn to adjust to them.

The post-encounter meetings contrasted with the initial team interaction. After the frontstage performance, the student team could “relax.” The backstage setting now allowed for a different kind of openness where students were more open about their uncertainties when returning backstage. Sharing this uncertainty and lack of knowledge with each other could imply a more laid-back backstage, where the team members were allowed to reveal their knowledge gaps openly.

## Discussion

The purpose of this study was to gain a better understanding of how interprofessional student teams interact with patients in interprofessional clinical placements.

Across cases and contexts, our findings indicate that patients are central to the teams’ collaborative processes; however, patients are not always sufficiently included in team-based encounters.

Undergraduate students in interprofessional learning arrangements change their attitudes and gain knowledge about other health professions (Barr et al., 2006). Reeves et al. (2012) found that undergraduates often reported changes in beliefs, knowledge, attitudes, and collaborative care after IP arrangements. Re-organization of practice and improvements in care delivery were less reported at an undergraduate level than in postgraduate studies (Reeves et al., 2012).

This study shows that interprofessional clinical arrangements enable students to learn with, from, and about each other. Students also learn that the patient encounter impacts their practice with the patient and within the team. When considering the expected learning outcomes for IPE on “the patient” (Thistlethwaite & Moran, 2010), students recognize that the patient's health issues and perspectives are also considered; however, partnering with the patient is not identified to a great extent. We believe the latter is an essential finding, especially considering that students were in their final undergraduate year, some only months away from graduating. One explanation may be related to the students´ traditional training, including mainly one-to-one interactions in uni-professional arrangements and interprofessional team-based encounters that were unfamiliar to the students. Thus, the students´ former experiences may have impacted how they interacted with each other and the patient. It is necessary to question if we would identify a more inclusive interaction with the patient if team-based encounters were the norm and the students were trained for this at an earlier point or on several occasions in their education.

The different interprofessional clinical arrangements where student teams interact with patients have the potential to train PCC. However, our study indicates that there is a potential for more explicitly talking about PCC both backstage and frontstage, with and without the patient. We consider the patient and their story central when students learn interprofessional collaboration because the patient is the outspring of the teams’ agendas and actions. We argue that students’ learning outcomes on collaboration would be impaired if the patient was lacking as the patient encounter in many cases triggered, e.g., professional knowledge sharing between students. Our analysis shows how adjusting preconceived pictures of patients helped the students express what competence was needed and showed how flexibility played a part in the learning arrangements. Findings from other studies on clinical placements support this finding and address the flexibility concerning how interprofessional students learn to communicate with each other (Howell et al., 2012). Even in the digital context, where teams were set and did not have the flexibility to invite other professions into their teamwork, the students discussed and problematized how their different professions could contribute to the patient’s situation.

Meeting patients forces adjustments in interprofessional students’ novice collaborative practices. In slight contrast to Reeves et al. (2012), our study shows a potential ability for undergraduates to re-organize and improve their care-delivery plans at least on a micro level, for the individual. We consider patient encounters a key for interprofessional student teams to learn with, from, and about each other and the patient as the meetings expand their perspectives on each other as professionals and their perspective on the patient.

Our study identified that patients were given space and a role by the interprofessional student teams; however, they still did not get the space they potentially could have had. As initially introduced, PCC includes patients’ holding expert knowledge of their own lives and health situations (Berwick, 2009). By involving the patient, personalized care and treatment can be obtained. Respect for the patients, caring for them on their terms, and being listened to, informed, and involved are emphasized (Epstein & Street, 2011). Including the patient as a partner can achieve democratic encounters that can contribute to better experiences, better diagnostic practice, and enhanced patient safety (Bleakley, 2014). Patients also find it positive to be present in teaching and supervision arrangements of health professional students where their own health concerns are addressed (Cheema et al., 2022).

Fox and Reeves (2015) argue that patient-centeredness may not favor all patients. They point to patients’ socio-economic status and how some patients may not be able to or even want to be partners in their healthcare decisions. They also exemplify how patients have been reprimanded by physicians when trying to take on the expert role and obtain more knowledge about their health issues. Our study shows that the patients’ narratives were not always heard and appreciated by the students. Some patients expressed in interviews that they did not feel they had the opportunity to tell their stories to the interprofessional students. Through an explicit focus on PCC in clinical IPE for undergraduates, a thematization of practical communication issues, power distribution between health professionals, and power distribution between health professionals and patients can occur.

Bleakley and Bligh (2008) claimed that modern medical education seems to refuse “the deliberate use of patients as the primary source for learning” (p. 90). They argue that educators still need a reminder that the patient is the primary concern of health and medical work (Bleakley & Bligh, 2008). Paradoxically, patient-centeredness is learned through the language and eyes of professional educators (doctors, nurses, etc.) and not from the patient (Bleakley & Bligh, 2008). In our study, the interactions between students and patients occurred mainly without supervisors. Where supervisors are available, they function as support and not gatekeepers for students’ interaction with patients. This is in accordance with what Bleakley and Bligh (2008) claimed as the optimal interaction between students and patients for learning patient-centeredness. However, patient-centeredness was not thematized directly in the student teams or by supervisors in our study; thus, we question whether students are aware of this aspect of learning in the IPE arrangement and if the thematization of patient-centeredness would cause other forms of interaction with the patients.

It may also be necessary for learning patient-centeredness that the patients were aware of the learning aspect; they may have “cut some slack” to the students, acknowledging that they were in a learning process and not fully trained. Marshall et al. (2012) explored patients’ views on PCC. They reported that being actively involved, health personnel being attentive, and feeling a connectedness between themselves and their care providers were important for patient-centeredness. Our findings show students’ attempts to actively involve the patient and attentiveness toward health aspects; however, the patient feeling connected may not be as evident.

As Kent et al. (2016b) found, there may be tension between students’ objectives of learning to become a health practitioner and learning teamwork and the patient's needs for a health consultation. For the patient, it may also become an internal tension of being an educator and a health care receiver. In our study, the patients expressed their views on the encounters based on their experience of receiving health care and being assessed; we interpret that they did not identify themselves as educators. This may have something to do with how patients are prepared for their encounter(s) with student teams and how they have been empowered to take a role as an educator, or at least reflected on what the students can learn from the unique meeting. It is reason to argue that patients must be empowered to request a patient-centered approach from healthcare providers and educate students about this, including what PCC means for them as individuals. Accordingly, students can incorporate this knowledge about the patient into their collaborative training.

Finally, the different experiences in clinical learning arrangements can be used as a momentum to trigger students’ reflection and to explore further what PCC might be in the various settings in which students and patients perform and interact. Patient-centeredness in interprofessional learning arrangements and interprofessional practice may be thematized with the patient in both the backstage and frontstage. Educators must encourage students to discuss this matter with patients, supervisors, and each other. And not least, patients must be empowered to participate in the PCC discussion and how this can be understood in the individual’s context.

These findings help clarify the interactions between interprofessional student teams and patients in clinical placements. We must explore how patients can contribute to educating students on interprofessional collaboration and patient-centeredness. Both a strength and limitation of this study are how the participant observation has a holistic approach; on one side the complexity of different encounters are captured, on the other side details may have been overseen. A more in-depth analysis of the discourse between students and patients may help elucidate this interaction. A second limitation is a minor focus on the supervisor’s role. While this study enhances our understanding of interprofessional student teams and patient-centeredness in patient interaction, further studies regarding supervision in such interprofessional clinical placements would be worthwhile.

## Reflexivity

The first author, CBJ, can serve as a researcher, educator, and registered nurse to hold both an emic and an etic position. For instance, CBJ had a prior relationship as a former nurse educator with some of the students in the first observation period. The research team balanced the possible emic view with an etic consideration of the data. Reflections on “participant reactivity” (Paradis & Sutkin, 2017), that is how a researcher´s presence impacts participants natural performance, were addressed by the research team. Participants reported in interviews that they mostly did not take notice of the researcher´s presence. We believe that using multiple data sources and reflecting together with participants about their interaction with each other strengthens the credibility of our study (Frambach et al. 2013).

## Data Availability

Research data are confidential and not accessible.
